# Tetherin Restricts Productive HIV-1 Cell-to-Cell Transmission

**DOI:** 10.1371/journal.ppat.1000955

**Published:** 2010-06-17

**Authors:** Nicoletta Casartelli, Marion Sourisseau, Jerome Feldmann, Florence Guivel-Benhassine, Adeline Mallet, Anne-Geneviève Marcelin, John Guatelli, Olivier Schwartz

**Affiliations:** 1 Institut Pasteur, Virus and Immunity Unit, URA CNRS 3015, Paris, France; 2 Plateforme de Microscopie Electronique, Institut Pasteur, Paris, France; 3 UPMC University Paris 06, and Laboratoire de Virologie, Hôpital Pitié-Salpêtrière, Paris, France; 4 Department of Medicine, University of California San Diego, La Jolla, California, United States of America; University of Geneva, Switzerland

## Abstract

The IFN-inducible antiviral protein tetherin (or BST-2/CD317/HM1.24) impairs release of mature HIV-1 particles from infected cells. HIV-1 Vpu antagonizes the effect of tetherin. The fate of virions trapped at the cell surface remains poorly understood. Here, we asked whether tetherin impairs HIV cell-to-cell transmission, a major means of viral spread. Tetherin-positive or -negative cells, infected with wild-type or ΔVpu HIV, were used as donor cells and cocultivated with target lymphocytes. We show that tetherin inhibits productive cell-to-cell transmission of ΔVpu to targets and impairs that of WT HIV. Tetherin accumulates with Gag at the contact zone between infected and target cells, but does not prevent the formation of virological synapses. In the presence of tetherin, viruses are then mostly transferred to targets as abnormally large patches. These viral aggregates do not efficiently promote infection after transfer, because they accumulate at the surface of target cells and are impaired in their fusion capacities. Tetherin, by imprinting virions in donor cells, is the first example of a surface restriction factor limiting viral cell-to-cell spread.

## Author Summary

Tetherin is a cell surface “restriction factor” that acts as an innate antiviral defense. Tetherin prevents newly produced particles of HIV-1 and other enveloped viruses from escaping the surface of infected cells. HIV-1 encodes the protein Vpu to counteract this host defense. We have studied here if HIV-1 particles trapped at the cell surface may be transmitted to neighboring uninfected cells. Direct transmission through cell-to-cell contacts is indeed an efficient means for viral spread. Virological synapses may be formed between infected donor cells and target cells, allowing rapid and massive transmission of viruses. We show that tetherin inhibits productive cell-to-cell transmission of Vpu-deleted HIV to target cells, and impairs that of wild-type virus. Tetherin accumulates with Gag at the contact zone between infected and target cells, but does not prevent the formation of virological synapses. With tetherin, viruses are then mostly transferred to targets as abnormally large patches that are impaired in their fusion capacities. These results represent the first example of a surface restriction factor limiting viral cell-to-cell spread, acting in donor cells, but inhibiting infection after transfer of viral material to novel recipient cells.

## Introduction

HIV and many other viruses move not only as free viral particles diffusing in the extracellular environment, but also directly between cells [1]. Cell-to-cell spread accelerates viral dissemination, and likely influences pathogenesis and immune evasion [1]. Various modes of cell-to-cell HIV transfer have been reported in culture. HIV-1 readily forms virological synapses (VS) at the interface between HIV-infected cells and targets. VS formation involves HIV Env-CD4-coreceptor interactions, and requires cytoskeletal rearrangements and stabilization of cell junctions by adhesion molecules [1], [2]. Other modes of retroviral cell-to-cell spread include polysynapses, which allow simultaneous transfer to multiple targets [3], filopodial bridges or thiner nanotube-like structures formed between infected cells and more distant targets [4], [5], and biofilm-like HTLV-I assemblies embedded in extracellular matrix components [6]. HIV dissemination through VS occurs within minutes and involves viral endocytosis in target cells [7]–[9]. Type-I interferons (IFN) inhibit partially HIV cell-to-cell transmission [10], but the interferon-induced protein(s) responsible for this inhibition are not characterized.

Tetherin (also known as BST-2, CD317 or HM1.24) is an interferon-induced protein recently identified as inhibiting the release of retroviruses and other enveloped viruses [11]–[17]. The non-structural Vpu protein of pandemic HIV-1 strains counteracts tetherin, by inducing its removal from the cell surface and its proteasomal and/or lysosomal-dependent degradation [11], [12], [18]–[23]. Some primate lentiviruses that do not encode Vpu may use Nef or Env to antagonize tetherin [24]–[28]. A few viruses (SIVcpz and SIVgor) also use Nef to down-regulate tetherin, although they contain Vpu genes [28]. Moreover, there are species-specific activities of Vpu and Nef in overcoming restriction by tetherin [24]–[29]. The mechanism of action of tetherin is partly understood. Tetherin dramatically inhibits the release of ΔVpu virions and moderately affects that of WT HIV [11], [12], [30]. In infected cells, tetherin colocalizes with Gag proteins [11], [12], and retains fully formed and mature viral particles at the cell surface [30], [31]. Tetherin is an integral membrane protein, with a short N-terminus located in the cytoplasm, which carries sorting signals for the endocytic machinery, and a glycosyl-phosphatidylinositol (GPI) anchor at the C-terminus [11], [32]–[34]. The protein is enriched in lipid rafts, which are sites of viral assembly and release [35], [36]. Tetherin is directly incorporated in budding virions as a parallel homodimer and restrains them at the cell surface [30], [31]. Tetherin binds to BCA2/Rabring7 to promote restriction [37]. Proteolysis of tetherin ectodomain releases virions retained on the cell surface, but cleavage of the GPI anchor does not [31]. Remarkably, an artificial tetherin-like protein, lacking sequence homology but mimicking its structure, recapitulated the antiviral activity [30]. The fate of membrane-tethered virions is not well known. A fraction of trapped virions is endocytosed by a BCA2/Rabring7-, Rab5a- and clathrin-dependent mechanism [38] [37] but a large part remains at the cell surface, forming aggregates [11], [12], [38].

Here, we asked whether the membrane-bound virions trapped by tetherin may be transmitted during intercellular contacts, and examined the impact of this restriction factor on viral cell-to-cell transmission and VS formation.

## Results

**Figure 1:**
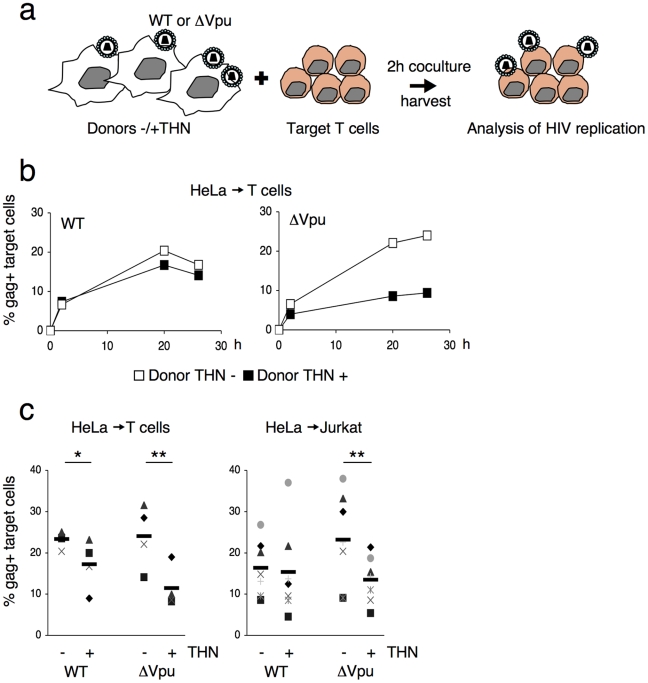
Tetherin reduces HIV cell-to-cell transmission. (**a**) Schematic representation of the cell-to-cell transfer assay. (**b**) HIV cell-to-cell transmission analyzed by flow cytometry. Hela donor cells expressing (black squares) or not expressing (white squares) tetherin (THN) were infected with WT (left panel) or ΔVpu (right panel) HIV. Cells were then cocultivated with target lymphocytes. The percentage of Gag+ cells in targets, at different time points is shown in this representative experiment. (**c**) Mean±sd (black line) of 4 and 7 independent experiment (20 h time point) with primary T cells (left) and Jurkat T cells (right) as targets, respectively. *p<0.05; **p<0.01 (Mann-Whitney test).

We analyzed the influence of Vpu on HIV cell-to-cell transmission. We cocultured for 2 h WT- or ΔVpu-infected cells with primary CD4+ lymphocytes, and then harvested the target lymphocytes. We then followed productive viral spread to lymphocytes by measuring by flow-cytometry the appearance of Gag+ cells, as outlined Figure 1a. We first used as donors HeLa cells, that constitutively express tetherin, or Hela-THN- cells, in which the protein was silenced (Figure S1a). Productive entry of viruses in HeLa or HeLa-THN- cells (which lack the CD4 receptor) was ensured by pseudotyping WT or ΔVpu virions with the VSV-G envelope. HeLa cells, with similar levels of infection (15–20% of Gag-expressing cells, as assessed by flow cytometry), were then cocultivated with CD4+ lymphocytes. WT HIV was efficiently transmitted to targets, with about 20% of lymphocytes expressing Gag after 18 h (Figure 1b). Nevirapine, a reverse-transcriptase inhibitor, significantly decreased the appearance of Gag in targets, confirming that the signal mostly originates from newly synthesized viral proteins, and not from capture of incoming virions (not shown). Transmission of WT HIV was slightly affected by tetherin. This confirmed that tetherin inhibition by Vpu is not absolute [12], [31] and likely depends on the relative levels of the two proteins. ΔVpu was transmitted from HeLa-THN- cells, but much less potently from HeLa cells (Figure 1b). A compilation of independent experiments, with lymphocytes from different donors, indicated that tetherin significantly decreased Gag expression in ΔVpu recipient cells (two-fold reduction) (Figure 1c). Similar results were obtained when Jurkat lymphoid cells were used as targets (Figure 1c). Thus, tetherin decreases HIV cell-to-cell transmission. Vpu counteracts this phenomenon. Noteworthy, the inhibitory effect of tetherin on ΔVpu was counteracted by transient transfection of a Vpu expression plasmid in donor HeLa cells, excluding the possibility that the expression of Vpu in targets may have biased the results (not shown).

**Figure 2:**
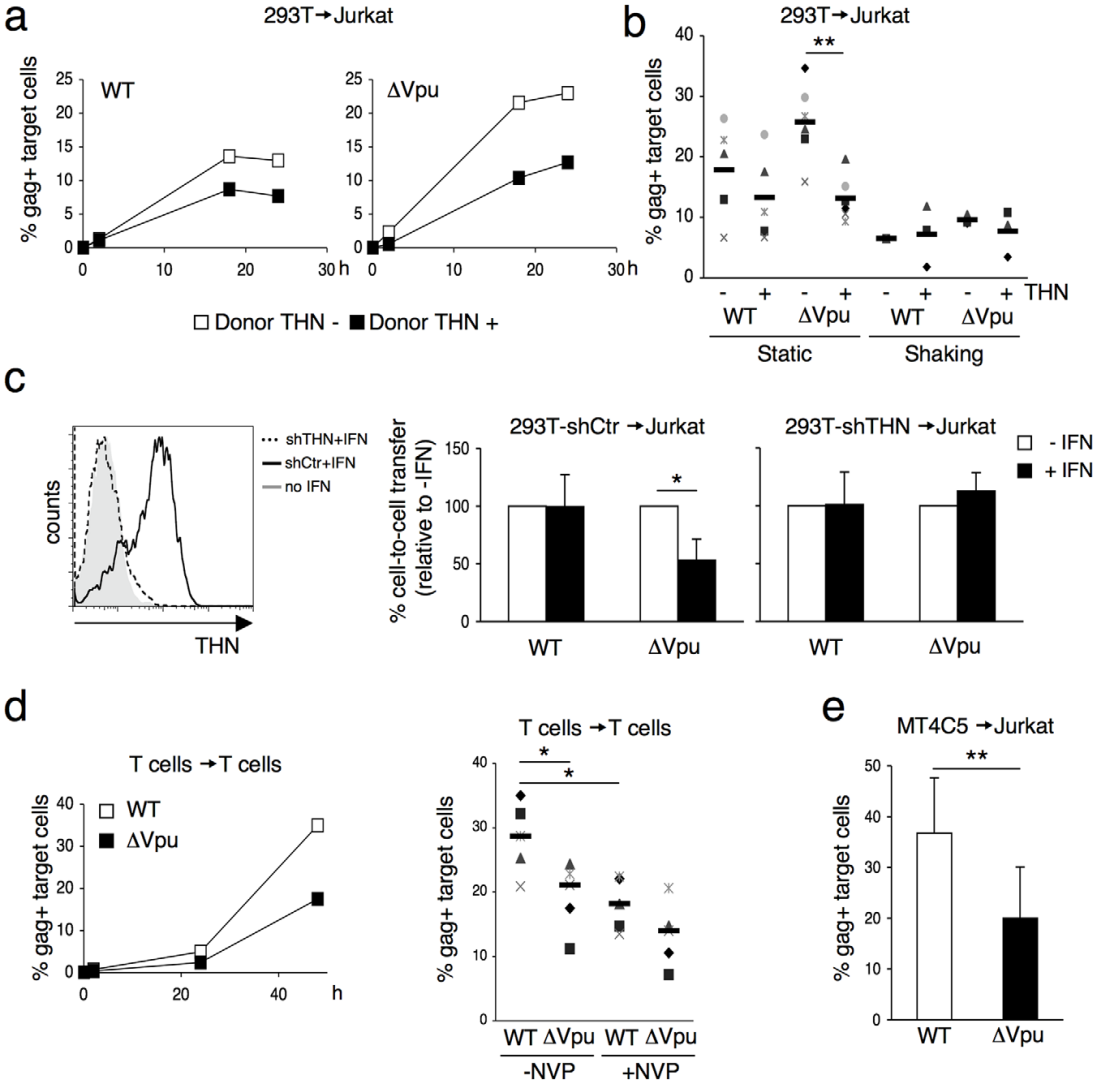
Tetherin reduces HIV cell-to-cell transmission from 293T and primary T cells. (**a**) Effect of transient expression of tetherin. 293T cells donor cells were cotransfected with WT (left panel) or ΔVpu (right panel) HIV proviruses, along with a control (white squares) or a tetherin expression plasmid. Cells were then cocultivated with target Jurkat cells for 2 h. The percentage of Gag+ cells in targets, at different time points after harvesting the targets, is shown in this representative experiment. (**b**) Mean±sd (black line) of 6 independent experiment (20 h time point) with Jurkat T cells as targets. When stated (in 3 out of 6 experiments), cocultures were gently shaken to inhibit intercellular contacts *p<0.05; **p<0.01 (Mann-Whitney test). (**c**) Tetherin expression in IFN-treated 293T cells stably expressing a control shRNA (continuous line) or an shRNA targeting THN (dotted line). Grey histogram: cells not treated with IFN. Cell-to-cell transfer using infected 293T cells as donors and Jurkat as targets (right panels). Mean±sd of 3 independent experiments is depicted. (**d**) HIV transfer between primary T cells. Donor lymphocytes were infected with WT (white squares) or ΔVpu (black squares) HIV. The percentage of Gag+ cells among targets is shown at the indicated times points in this representative experiment. Right: mean (black line) ±sd of 4 independent experiments. Targets were treated or not with Nevirapine (NVP), a reverse transciptase inhibitor, after coculture. (**e**) Compilation of 3 independent experiments using MT4C5 T cells as donors and Jurkat as targets. Data are mean ±sd. **b, c, e**: 20 h time point; **d**: 48 h time point. *p<0.05; **p<0.01 (Mann-Whitney test).

We then used 293T cells as donors, since they do not naturally express tetherin. We examined if transient expression of tetherin inhibited viral cell-to-cell spread. To this end, 293T cells were cotransfected with WT or ΔVpu HIV proviruses, along with a control or a tetherin expression plasmid. An amount of 100 ng of tetherin plasmid was selected, since it potently inhibited release of ΔVpu, without affecting that of WT HIV (Figure S1b). Upon coculture of transfected cells with Jurkat cells, ΔVpu transmission to target lymphocytes was decreased by tetherin, whereas WT HIV was minimally impaired (Figure 2a). A compilation of 6 independent experiments confirmed a significant reduction (two-fold) of ΔVpu transmission from tetherin positive cells, when compared to negative cells (Figure 2b). We next evaluated the contribution of cell-free viral particles to the productive infection of target cells. We previously reported that a gentle agitation of cocultures inhibits HIV spread through direct cell contacts without impairing infection by free virions [39]. Shaking cocultures of 293T donor cells and Jurkat target cells strongly inhibited the appearance of Gag+ cells in targets, irrespectively of the presence of tetherin or Vpu in donors (Figure 2b). Therefore, under these experimental conditions, most of productive viral transmission occurs through intercellular contacts. The contribution of cell-free virions is minimal.

To describe further the impact of tetherin on HIV cell-to-cell spread, we transfected different amounts of tetherin expression plasmids (Figure S1b). At low amounts (20 ng of transfected DNA), ΔVpu release in supernatants was inhibited, without obvious effects on cell-to-cell transmission. With high levels of tetherin (200 ng), ΔVpu release and transmission were both restricted. This was also the case for WT HIV. Therefore, as previously reported for viral release [11], [12], [30], the effect of tetherin on cell-to-cell spread is dose-dependent. The anti-tetherin activity of Vpu is not absolute, and tetherin inhibits more easily viral release than cell-to-cell transmission.

We then asked whether tetherin, when induced by type-I interferon (IFN), restricts intercellular viral spread. We generated 293T cells that carry an shRNA against tetherin (293T-shTHN) or a control shRNA (293T-shCtr). Flow-cytometry indicated that IFN induced tetherin in 293T control cells, but not in 293T-shTHN cells (Figure 2c). Upon IFN treatment, cell-to-cell spread of ΔVpu was significantly impaired in control cells (two-fold decrease), and occurred normally in tetherin-silenced 293T cells (Figure 2c). Therefore tetherin is the major interferon-induced protein impairing HIV cell-to-cell transfer in this experimental setting.

It was important to determine whether tetherin also inhibits lymphocyte-to-lymphocyte viral transfer. Primary lymphocytes, naturally expressing tetherin (not shown), were infected with WT or ΔVpu HIV, and were then cocultivated with uninfected target lymphocytes, where the appearance of Gag+ cells was measured over time. With WT HIV, about 30% of Gag+ targets were detected at 48 h. This signal was only partly inhibited in the presence of Nevirapine, suggesting that it corresponds to a mix of newly synthesized Gag proteins and incoming viral materials bound to targets (Figure 2d). ΔVpu was significantly less transmitted to recipient lymphocytes. T. Interestingly, in the presence of Nevirapine, the % of Gag+ target cells was not significantly different for WT and ΔVpu (Figure 2d), suggesting that uptake of incoming viral materials by targets is not inhibited by tetherin. This dissociation between viral uptake and subsequent infection of target cells is studied further below. A decrease of ΔVpu productive transmission was observed when various T cell lines (MT4C5, Jurkat, or CEM) all expressing tetherin, were used as donors or targets (not shown and Figure 2e for an example of viral transfer from MT4C5 to Jurkat cells). To directly assess the role of tetherin in lymphocytes, we generated CEM cells in which expression of the protein was silenced (CEM-THN-, with about 90% of Tetherin down-regulation, Figure S2a). CEM-THN- transmitted more efficiently ΔVpu to target lymphocytes than parental CEM cells (Figure S2b). Altogether, these results show that tetherin significantly reduces HIV cell-to-cell transmission from various primary and permanent cell types (HeLa, 293T, and lymphocytes).

**Figure 3:**
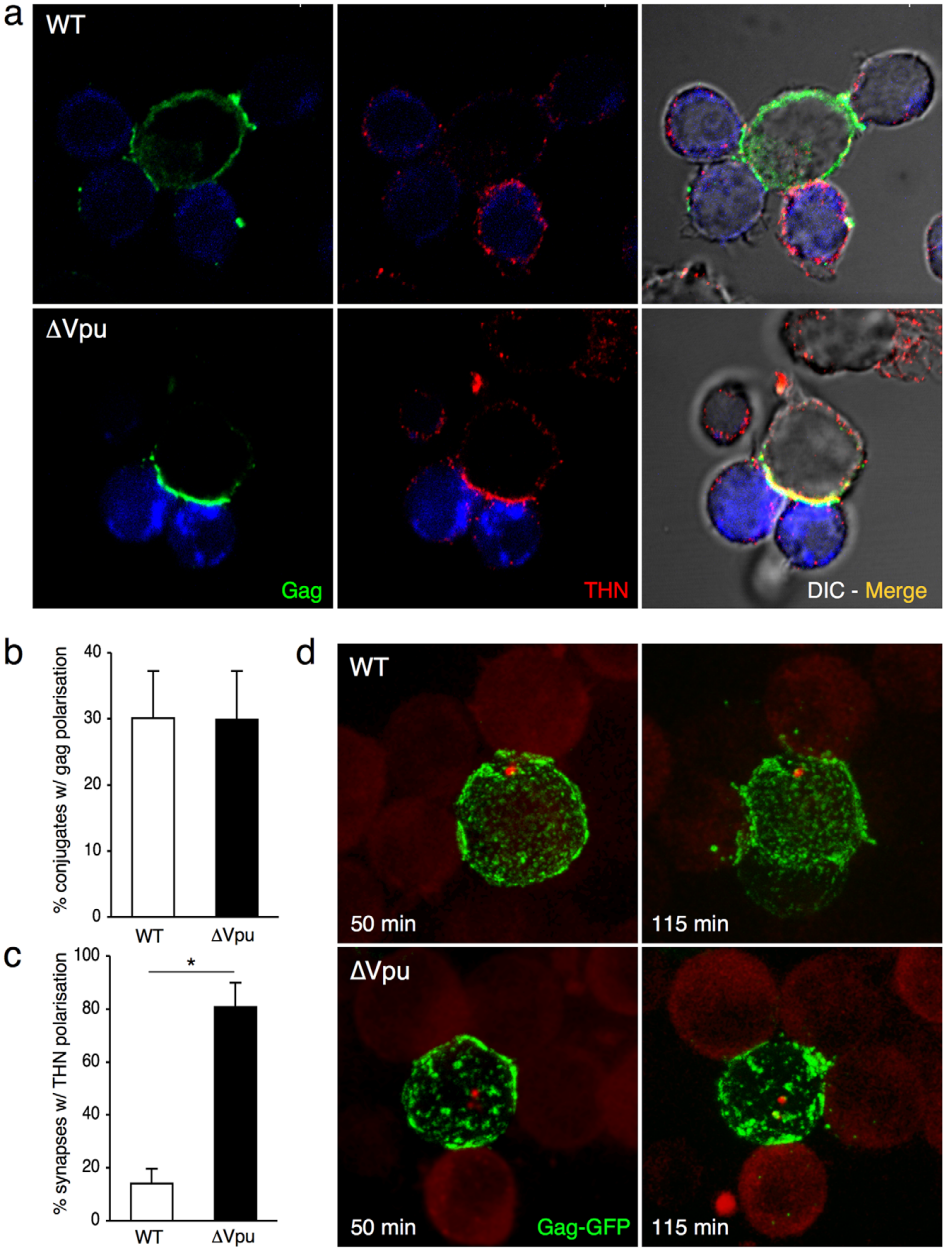
Tetherin accumulates with Gag at the virological synapse. (**a**) Localization of Gag and tetherin at the virological synapse. WT or ΔVpu HIV-infected MT4C5 cells were mixed with far-red-dye labeled Jurkat recipients (blue) for 1 h, and stained for HIV-1 Gag (green) and tetherin (red). Representative images are shown. (**b**) Quantification of infected MT4C5 cells displaying Gag clustering at the junction zone with targets. Data are mean±sd of 5 independent experiments (with a least of 650 Gag+ cells analyzed for each condition). (**c**) Quantification of virological synapses displaying tetherin clustering at the junction zone. Data are mean±sd of 5 independent experiments (with a least of 200 synapses analyzed for each condition). (**d**) Live video-microscope imaging of cell-to-cell transfer. Jurkat cells transfected with WT or ΔVpu HIV-GagGFP were mixed with actin-RFP expressing Jurkat targets and analyzed. A 3D image was acquired every 20 seconds for 2 hours. 2D maximum-projection of the images is shown Elapsed time after mixing is indicated. Corresponding sequences are supplementary Videos S1 (HIV-GagGFP WT) and S2 (HIV-GagGFP ΔVpu). *p<0.05 (Mann-Whitney test).

Which step of viral spread does tetherin alter? We examined whether the protein affected VS formation in lymphocytes. We measured the recruitment of Gag proteins at the contact zone between donors and recipients, a hallmark of VS formation [1]. Lymphocytes (MT4C5 T cell line) were infected with Vpu positive or negative viruses. With ΔVpu, the cell surface Gag signal was generally more intense than with WT, and appeared mostly as large patches of fluorescence, reflecting the trapping of virions (Figure S3a). In non-infected MT4C5 cells, tetherin was found at the cell periphery and in intracellular compartments (Figure S3a), likely corresponding to the Golgi or endosomal network [11], [12], [32]. As expected, tetherin colocalized with Gag at the surface of ΔVpu-infected cells, and was down-regulated in WT-infected cells (Figure S3a). Infected cells were then incubated for 1 h with recipient lymphocytes (Jurkat cells stained with Far Red dye). The percentage of Gag+ donor cells forming conjugates with Far Red+ cells was similar with WT and ΔVpu viruses (not shown). About 30% of conjugates displayed a polarization of Gag at the junction, irrespectively of the presence of Vpu (Figure 3b). Interestingly, with ΔVpu, the large Gag-containing patches accumulated at the contact zone (Figure 3a). We then investigated the distribution of tetherin in conjugates of infected cells and targets. The protein colocalized with Gag at the synapse in about 80% of conjugates with ΔVpu, whereas it was much less present in WT-induced synapses, probably as a direct consequence of Vpu-mediated removal of tetherin from the cell surface (Figures 3a,c). Tetherin also accumulated at the intercellular zone when tetherin-negative Jurkat cells were used as targets (Figure S3b), strongly suggesting that molecules found at the VS originated from donors, without requiring the presence of the antiviral protein in targets. Tetherin enrichment was not detected with control non-infected donors mixed with targets (not shown). Altogether, these results show that tetherin does not prevent Gag polarization and VS formation, and accumulates with Gag proteins at the junction zone.

We next visualized the spatio-temporal events leading to viral transfer in living cells. We used an infectious HIV-GagGFP virus [3] and its Vpu-deleted counterpart. Jurkat cells producing WT and ΔVpu HIV-GagGFP were cocultivated with targets that expressed a red fluorescent protein (RFP)-actin, and images were acquired every 20 s for 2 h. As previously reported [3], [8], virological synapses or polysynapses readily formed with WT HIV, illustrated by Gag polarization at the junction and subsequent transfer (Video S1 and Figure 3d). In donor cells, ΔVpu HIV-GagGFP often appeared as patches which were larger than those observed with its Vpu-positive counterpart (Video S2 and Figure 3d), likely reflecting the activity of tetherin [11], [12]. Time-lapse analysis showed that the large patches of Gag proteins originated from all regions of the plasma membrane and gained access to intercellular contact zones (Video S2 and Figure 3d). Both WT and ΔVpu viral materials from donor cells were then in part transferred to recipient cells (Videos S1 and S2).

**Figure 4:**
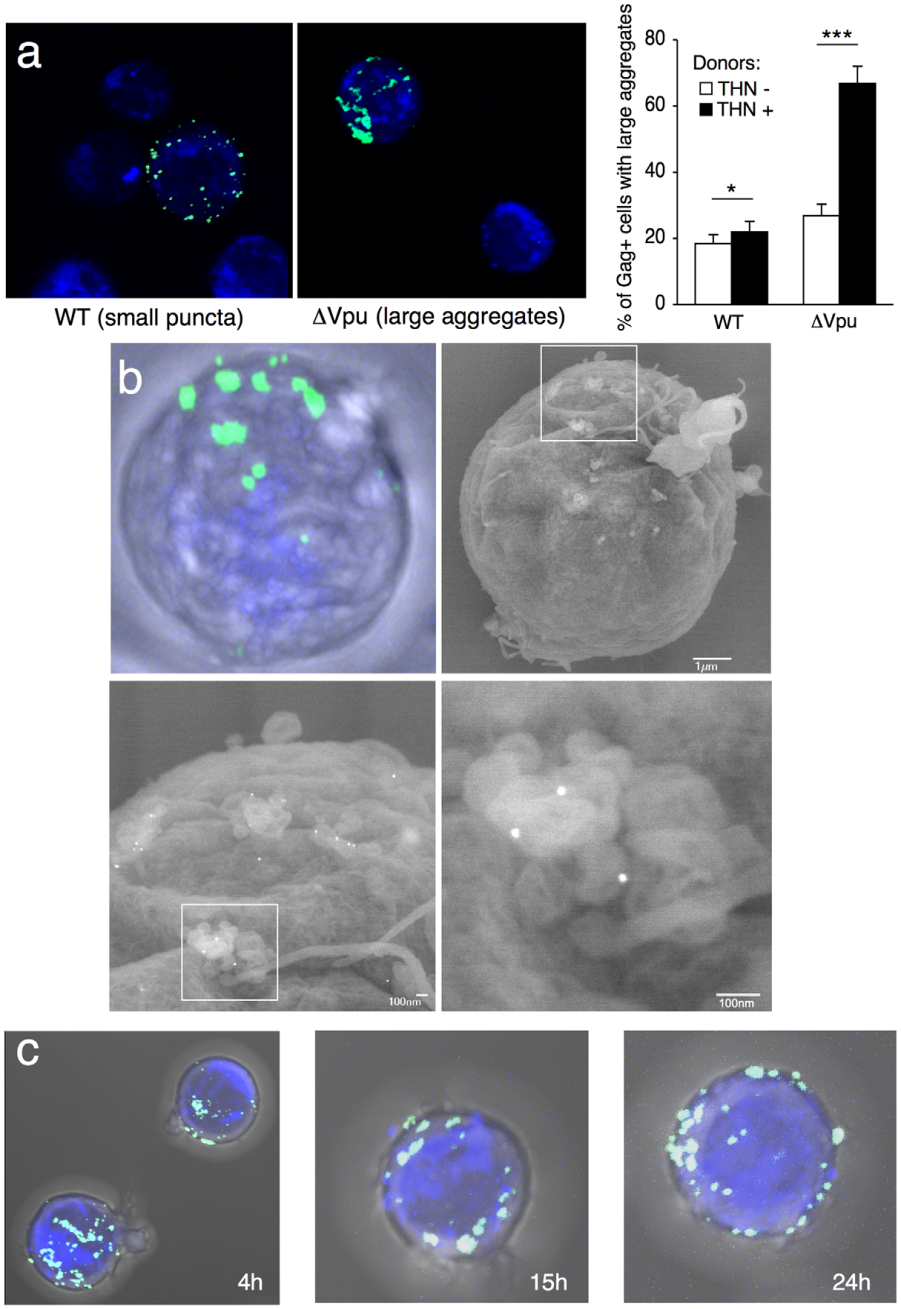
Distribution of transferred WT or ΔVpu viruses on target Jurkat cells. (**a**) Gag signal on target cells. Jurkat cells labelled with far-red dye (blue) were harvested after 2 h of contact with WT or ΔVpu HIV-Gag-GFP transfected HeLa. Representative images are shown. In the right panel, quantification of target cells displaying large aggregates of Gag-GFP proteins after 2 h of coculture with WT or ΔVpu HIV-transfected HeLa (white) or HeLa-THN- cells (black). Data are mean±sd of 9 independent experiments (with a least of 2000 Gag+ cells analyzed for each condition). Similar results were obtained with parental WT or ΔVpu viruses lacking GFP, and staining with Gag mAbs (not shown) (**b**) Correlative electron microscopy analysis of Jurkat targets after coculture with ΔVpu HIV-GagGFP transfected Hela cells. Viral aggregates appearing as green spots in the IF image were visualized by SEM. Cells are stained with anti-Env MAb coupled to 20 nm-gold particles (appearing as white dots). (**c**) Long-lived Gag patches on target cells. Jurkat cells were harvested after 2 h of contact with ΔVpu HIV-Gag-GFP transfected HeLa and incubated at 37°C in presence of nevirapine. Representative images of gag signal at different time points after coculture are shown.*p<0.05; ***p<0.005 (Mann-Whitney test).

**Figure 5:**
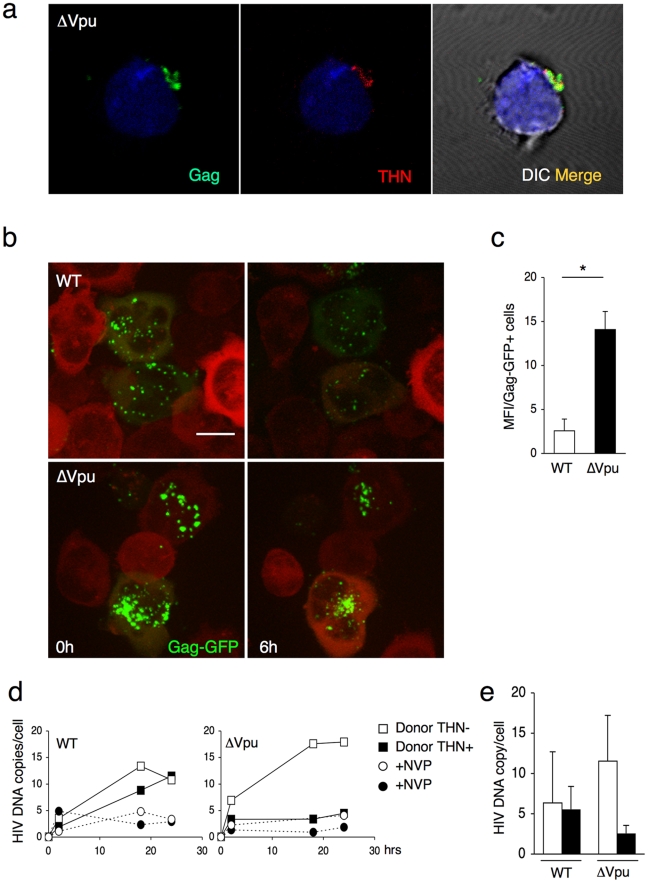
Tetherin promotes transfer of large viral patches and inhibits productive infection. (**a**) Distribution of Gag and tetherin. Jurkat target cells were stained, after 1 h of contact with ΔVpu-infected MT4C5, for Gag (green) and tetherin (red). A representative image from 5 independent experiments is shown. (**b**) Live video-microscope imaging of transferred virus on target cells. Actin-RFP Jurkat target cells, incubated for 4 hours with HIV-GagGFP WT or ΔVpu transfected Hela donor cells, were harvested and plated on fibronectin-coated micro-dish. Images were taken every 5 minutes for 10 hours. Elapsed time after the beginning of acquisition is indicated. 2D maximum-projection of the images is shown. The scale bar represents 10 µm. Corresponding sequences are available as supplementary Videos S3 (HIV-GagGFP WT) and S4 (HIV-GagGFP ΔVpu). (**c**) Quantification of WT or ΔVpu Gag-GFP fluorescence on Jurkat target cells after 2 h incubation with Hela donor cells. The virus-associated fluorescence was measured for each condition on at least 50 individual cells. The results are expressed as the mean fluorescence intensity of viral aggregates per Gag-GFP positive cell ± sd. Results are representative of 3 independent experiments. *p<0.05 (Mann-Whitney test). (**d**) Time course analysis of HIV DNA synthesis in Jurkat cells by qPCR. A representative experiment is shown on the left. Target cells were treated or not with nevirapine (NVP), a reverse transcriptase inhibitor, after the coculture. (**e**) Mean ±sd of 3 independent experiments is shown on the right (20 h time point).

What is the behavior of WT and ΔVpu viruses after their transfer to target cells? Infected HeLa cells were cocultivated with Jurkat cells, targets were harvested after 2 h, and Gag distribution was examined. We readily distinguished two types of Gag staining after transfer, the first corresponding to small and discrete puncta, and the second associated with large aggregates of Gag proteins (Figure 4a). These two viral species mirrored those observed in donor cells. WT HIV was mostly transferred as small clusters, whereas ΔVpu appeared as large aggregates in 70% of the targets (Figure 4a). The number of ΔVpu-infected cells displaying large clusters was strongly reduced when HeLa-THN- were used as donors (Figure 4a). To document further these large Gag-positive bundles in target cells, we followed the localisation of ΔVpu HIV-GagGFP on targets by correlative microscopy analysis. This technique combines fluorescence and scanning electron microscopy of the same samples over a wide range of magnification. The Gag signal corresponded to an agglomeration of viral-like particles (VLPs), each with a size of about 100 nm, assembled as large clusters (Figure 4b). Immunogold staining revealed that these VLPs were decorated with HIV Env+ dots, and likely corresponded to HIV-1 virions (Figures 4b and S4). These VLPs were not visible in non-infected cells (not shown and [3]). Additional immunofluorescence stainings on target cells confirmed a colocalization of Gag and Env (Figure S5a). Moreover, target cell membrane labelling with cholera toxin, a raft marker, suggested that these Gag clusters accumulated at the surface (Figure S5b). These large patches were still observed 15 or 24 h after harvesting the targets, and are thus relatively long-lived (Figure 4c). The conglomeration of Gag in recipient Jurkats similarly occurred after coculture with ΔVpu-infected lymphocytes (Figure 5a), and is thus not due to the use of HeLa as donors. Furthermore, these large aggregates were positive for tetherin (Figures 5a and S5c). The tetherin signal originated from donors, since it was detected when using tetherin-negative Jurkat recipients (Figure 5a). Therefore, tetherin is transferred along with HIV particles to recipient cells. These observations are consistent with the incorporation of tetherin into virions [30], [31].

Productive cell-to-cell transfer of the R5 tropic AD8ΔVpu strain to MT4-CCR5+ cells was also inhibited by tetherin (Figure S6a). With AD8ΔVpu, the large characteristic patches of Gag-positive material were also readily detected in target CCR5-negative Jurkat cells and CCR5+ primary CD4+ lymphocytes (Figure S6b), and not in a CD4-negative Jurkat subclone (not shown). The results suggest that transfer of these viral patches requires CD4 binding but not coreceptor expression in recipient cells. This event, however, did not lead to productive infection in the absence of CCR5 (not shown). These results also demonstrate that tetherin can restrict intercellular spread of X4 and R5 viruses.

We further documented by real-time imaging the fate of viruses after their transfer to targets. After 2 h of coculture with WT and ΔVpu HIV-GagGFP producer cells, Jurkat cells were harvested and monitored for up to 6–8 h (Figure 5b and Videos S3 and S4). At time zero post coculture, ΔVpu viral aggregates were apparently larger, and more numerous than WT virions. A standardized quantification demonstrated a 7-fold increase in the fluorescent signal per cell with ΔVpu (Figure 5c). This confirmed that the impaired productive infection of targets did not result from a reduced transfer of viral material. The fate of incoming viral particles was apparently different with WT and ΔVpu. In the presence of Vpu, the punctate fluorescent signals decreased in number and intensity overtime. In addition to signal quenching, this decrease may reflect viral detachment, endocytosis, degradation or fusion events (Figure 5b and Video S3). In the absence of Vpu, the large patches were apparently stable, some of them remaining visible at the plasma membrane after 6–8 h (Figure 5b and Video S4).

We then assessed the early events of viral replication in targets by quantifying viral DNA synthesis. HeLa and HeLa-THN- cells, infected with WT and ΔVpu HIV, were cocultivated with Jurkat cells for 1 h. Target cells were then harvested, further incubated at 37°C and analyzed as a function of time by qPCR for the presence of reverse transcription (RT) products. Nevirapine was included as a control to ensure that the detected PCR products were the result of proviral DNA neosynthesis (Figures 5 d, e). With WT, we observed an increase of RT products overtime, reaching about 10 copies per cell at 24 h after infection (Figures 5 d,e). Viral DNA synthesis was similar when tetherin positive and negative cells were used as donors (Figures 5 d,e). The situation was different with ΔVpu. With tetherin-negative donors, viral DNA synthesis occurred efficiently, reaching 10–15 copies at 24 h, which is even slightly higher than levels observed with the WT virus (Figures 5 d,e). Tetherin drastically reduced the appearance of RT products, which barely exceeded background levels observed in Nevirapine-treated cells. Therefore, tetherin, when expressed in donor cells, imprints the virus, resulting in a strong decrease (5 fold reduction) of viral DNA synthesis after viral transfer to targets.

**Figure 6:**
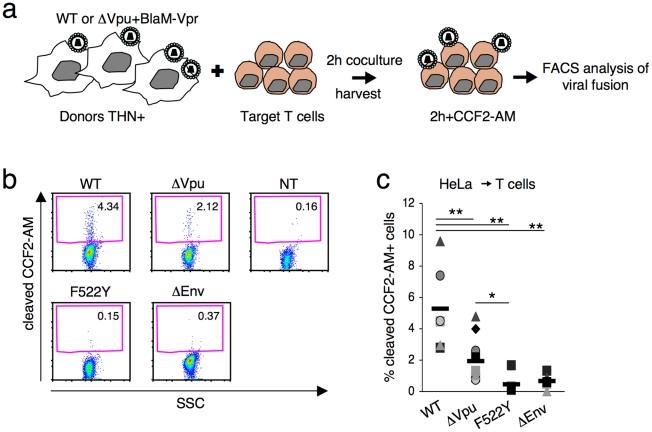
Tetherin reduces fusion after viral transfer to target cells. (**a**) Schematic representation of the viral fusion assay after cell-to-cell transfer. (**b**) HIV fusion analyzed by cytometry. Jurkat T cells were cocultivated with donor Hela cells for 2 h, harvested and incubated at room temperature for 2 h with CCF2-AM. Viral fusion was evaluated by measuring the percentage of cells positive for cleaved CCF2-AM. A representative experiment is shown. (**c**) The percentage of cleaved-CCF2-AM+ cells in 7 independent experiments is shown along with the mean value (black line). *p<0.05; **p<0.01 (Mann-Whitney test).

Our results indicate that tetherin impairs an early step of the viral cycle. We hypothesized that the fusion ability of the viral aggregates could be reduced. We adapted a cell-free virion-based assay [40] to analyze viral fusion after cell-to-cell transfer. This assay involves the use of viruses containing a beta-lactamase-Vpr (BlaM-Vpr) protein chimera (see experimental outline Figure 6a). After 2 h of coculture with infected cells, target cells are harvested. The successful cytoplasmic access of Blam-Vpr as a result of fusion is monitored by the enzymatic cleavage of CCF2-AM, a fluorogenic substrate of beta-lactamase loaded in targets. We used as donor HeLa cells endogenously expressing tetherin, and producing WT and ΔVpu HIV. Fusion of the wild-type virus was readily detected, with more or less 5% of target Jurkat harbouring cleaved CCF2-AM (Figures 6b,c). There was a significant (2.7 fold) decrease of fluorescent targets with ΔVpu (Figures 6b,c). We used as control donors cells producing an Env-deleted (ΔEnv) or a fusion-defective HIV (F522Y mutant, that carries a point mutation in Env abrogating fusion but retaining CD4 binding [41], [42]). None of these two mutants scored positive (Figures 6b,c), confirming that the assay detects viral fusion, and not virion endocytosis [40]. Furthermore, a gentle shaking of the cocultures inhibited the appearance of cleaved CCF2-AM+ target cells (Figure S7) strongly suggesting that cell-free viral particles play a minor role in this viral fusion assay. Altogether, our results demonstrate that tetherin impairs viral fusion and subsequent productive infection of target cells.

## Discussion

We show here that tetherin inhibits HIV cell-to-cell spread through an unexpected mechanism. With tetherin, virions are known to be trapped at the cell surface [11], [12], and to form aggregates. When infected cells harbouring these structures encounter uninfected cells, viral aggregates are routed to intercellular junction zones, and VS are formed quite normally. Tetherin also accumulates at the VS. This leads to the transfer of large aggregates of viral material to target lymphocytes. Scanning electron microscopy and immunofluorescence analysis demonstrated that aggregates reaching target cells are composed of viral particles stuck together, and likely incorporating tetherin in their membrane [30], [31]. The subsequent steps of viral replication are impaired. The viral conglomerates are able to move or surf at the surface of target for hours, as visualized by real-time imaging, but they lead to low levels of viral DNA synthesis. These results demonstrate dissociation between the physical “transfer” of viral materials to targets, which is not affected by tetherin and the subsequent infection, or “productive transmission” [43], which is blocked by the restriction factor.

We further show that the fusion process itself, leading to access of incoming viral material to the cytosol of target cells, is inhibited. Viral fusion is known to occur at the cell surface, or more efficiently after viral endocytosis [8], [44]. Our results strongly suggest that these clusters of viral particles do not fuse at the cell surface and/or are not adequately internalized. It is also noteworthy that both incoming virions and newly synthesized viral proteins generate the Gag signal in targets, when measured by flow cytometry. The proportion of these two signals varies when tetherin is present or absent in donor cells. This likely explains the modest but significant decrease (two-fold reduction) in the appearance of Gag+ cells induced by tetherin. Measuring viral infectivity after transfer by following viral DNA synthesis underscored a more marked inhibitory activity of tetherin (five-fold decrease).

How does tetherin act? We report here that the antiviral protein is necessary in donor cells, and not in targets (not shown), to block productive cell-to-cell transfer. Moreover, in recipient cells, the presence of CD4 (and not that of HIV coreceptors) is necessary to promote transfer of viral aggregates. The strength of virus trapping, which likely relies on non-covalent interactions [31], is thus not sufficient to prevent viral transfer through Env/CD4 interactions. Tetherin has been recently demonstrated to act directly on viral release by infiltration of viral membranes [30], [31]. This infiltration likely explains our observation that tetherin is co-transferred with particles. One can hypothesize that tetherin not only physically tethers virions together, but also interferes, either directly or indirectly, with a post-binding event. The packed accumulation of virions may prevent fusion or endocytosis, for instance by sterically blocking the function of Env. Tetherin itself, when associated with viral membranes, may additionally impair the ability of Env glycoproteins to mediate fusion. Alternatively, tetherin, as a GPI-anchored raft protein, might trigger the transfer of raft-associated cellular components that affect viral infectivity. These non-mutually exclusive possibilities will require further investigations. Noteworthy, we observed here that in the absence of tetherin, ΔVpu is slightly but reproducibly more transmitted than WT HIV. This may in part explain why Gummuluru *et al* selected an HIV mutant lacking a functional Vpu protein in an assay aimed at identifying fast-growing strains [45]. Vpu exerts diverse activities, and for instance down-regulates CD4 expression on infected cells. The global impact of Vpu on viral fitness is likely the consequence of tetherin-dependent and independent effects.

Our results show that tetherin significantly impairs HIV cell-to-cell transmission, which is a major means of viral replication in culture systems [39]. The inhibition of productive cell-to-cell transfer is directly linked to the trapping of virions at the surface of donors. Tetherin provides a physical link between lipid rafts and the apical actin network in polarized epithelial cells [33]. It will be of interest to determine further the role of rafts and of the actin cytoskeleton, which both regulate HIV cell-to-cell transfer [1], in the effects of tetherin.

Tetherin is able to inhibit the release of a variety of enveloped viruses, including other retroviruses (alpha-, beta- and delta-retrovirus, lentivirus, spumaretrovirus) and filoviruses (Marburg and Ebola viruses) [13], [14]. Most of viral species also spread through direct cell-to-cell spread [1]. HTLV-I is of special interest, since this virus is barely released from the cell, and replicates primarily by direct cell-to-cell transfer. Tetherin silencing enhances HTLV-I release [13]. In HTLV-I infected cells, large viral assemblies are present at the plasma membrane. These aggregates, termed viral biofilms, include components of the extracellular matrix, are positive for tetherin, and keep infectious potential after detachment [6]. Our results suggest that HIV aggregates and HTLV-I biofilms may impact differently viral infectivity and fate, since HIV aggregates are poorly infectious, much less than “individual” virions. Whether ΔVpu virions may be embedded in biofilm-like structures, together with components of the extracellular matrix, will require further investigation. It would be also of interest to determine whether these HIV aggregates, detached from donor cells for instance by a mechanical or chemical treatment, are effected in their fusion and infectivity capacities.

The physiological role of tetherin is not fully understood. This protein is induced by IFN or other stimuli in some cells including T lymphocytes, whereas it is constitutively expressed on other cell types like epithelial cells or plasmacytoid DCs. It is thus tempting to speculate that tetherin will act as a broad inhibitor of intercellular spread of various viruses in diverse cell types. Moreover, several viruses have evolved tetherin antagonists. It will be worth examining the role of these viral proteins during viral transmission.

In summary, we have demonstrated here that tetherin is an innate restriction factor limiting HIV cell-to-cell spread. This IFN-inducible protein acts through an original mechanism, by imprinting viruses in donor cells, and significantly reducing their infectious potential once they have been transferred to target cells.

## Materials and Methods

### Cells

Jurkat (clone 20), CEM and MT4C5 T lymphoid cells, Hela, and 293T cells were grown as described [3]. Primary CD4+ T cells were purified from human peripheral blood by density gradient centrifugation (Lymphocytes separation medium, PAA) followed by negative immunomagnetic selection (Miltenyi). About 98% of cells were CD4+CD3+. For activation, primary T cells were treated with phytohemagglutinin (PHA) (1 µg/ml) for 24 h at 37°C and then cultured in interleukin 2 (IL-2)-containing medium (50 IU/ml). Hela, 293T and CEM cells were electroporated (BioRad Gene pulser) with pRS-TI357703, coding for a 29-mer shRNAs targeting BST2 mRNA, or pRS expressing an off-target control (OriGene) [12]. Following electroporation, stable cell lines were generated by puromicyn selection (1 µg/ml). Resistant cell lines were then maintained in puromycin. Our Jurkat cells are naturally heterogenous in surface expression of tetherin. We directly sorted tetherin-negative cells from the parental population.

### Virus, infections and transfections

Virus stocks were prepared by transfection of 293T cells as described [3]. Cells were infected with the X4 HIV-1 strains NL4-3 or the NL4-3ΔVpu (referred to as HIV or HIVΔVpu), or when stated with the R5 strains AD8 WT and ΔVpu (a kind gift of Klaus Strebel) [46]. For infection of Hela and 293T cells, viruses pseudotyped with the vesicular stomatitis virus G protein were used to allow viral entry in absence of receptor expression. Infection was monitored by flow cytometry [39]. For HIV-GagGFP experiments, a Vpu-deleted version of pNLC4-3^EGFP^ [47] was a kind gift of Fabrizio Mammano. The EcoRI-BamHI fragment (containing *env* and *vpu*) of pNL4-3ΔVpu [46] was inserted in place of the EcoRI-BamHI fragment in pNLC4-3^EGFP^. HeLa cells were co-transfected with pNL4-3 and pNLC4-3^EGFP^ proviral vectors, or their Vpu deleted counterparts, by lipofection (Metafectene, Biontex) following manufacturer's instructions. 1 µg of each proviral vector was used to transfect 10^6^ cells.

### Analysis of cell-to-cell HIV transfer by flow cytometry

Infected or transfected Hela or 293T donor cells were plated in 24 well plates at a final concentration of 0.15×10^6^/ml. Two days later, when about 15–20% of the donor cells were Gag+, target cells were added to donor cells at a final concentration of 2×10^6^/ml in a final volume of 250 µl/well. Target cells were prelabelled with CellTrace Far Red DDAO-SE dye (1 µg/ml; Molecular Probes) for 10 min at 37°C. When stated, cocultures where gently shaken to inhibit intercellular contacts, as described [39]. After 2 hours of coculture, target cells were harvested, washed, and incubated at 37°C. At the indicated time points, cells were stained for intracellular Gag expression as described above and analyzed by flow cytometry. When stated, the reverse-transcriptase nevirapine (NVP 25 nM) was added 0.5 h before coculturing and maintained during the assay. With primary CD4+ T cells or T cell lines donors, the cell-to-cell transfer assay was conducted as previously described [39].

### Quantitative PCR of HIV DNA in target cells

Total DNA was extracted from target Jurkat cells using QIAamp DNA mini kit (QIAGEN, Courtaboeuf, France). Total HIV-1 DNA, including integrated and unintegrated HIV-DNA, was quantified in Jurkat cells by real-time PCR (amplification of a LTR region) [48].

### Correlative light-scanning electron microscopy (SEM)

Jurkat cells harvested after coculture with HeLa cells expressing HIV-Gag-GFP viruses were loaded on cell-locator glass-bottom dishes (MatTek Corporation) coated with poly-lysine. Cells were fixed in 4% PFA/0.1% Glutaraldehyde and prepared for correlative light-scanning electron microscopy (CL-SEM). Specific areas were imaged and localized with high resolution on the cell-locator glass-bottom dishes by using a Zeiss LSM510 microscope. Z series of optical sections were performed at 0.2 µm intervals. For subsequent SEM analysis, the same cells were refixed with 4% PFA for 1 hour. Immuno-gold labeling of HIV envelope (gold particles: 20 nm) was performed with anti-Gp120 mAb (110.H, Hybridolabs, Pasteur). Cells were fixed in 2.5% GA in 0.2 M cacodylate buffer (pH 7.2) overnight at 4°C, then washed for 5 minutes three times in 0.2 M cacodylate buffer (pH 7.2), post-fixed for 1 hour in 1% osmium, and rinsed with distilled water. Cells were dehydrated through a graded series of ethanol followed by critical point drying with CO_2_. Dried specimens were sputter-coated twice with carbon with a gun ionic evaporator PEC 682. The coordinates of the correlative cells imaged with fluorescent microscopy were recovered in a JEOL JSM 6700F field emission scanning electron microscope operating at 7 kV.

### Immunofluorescence and flow cytometry analysis

For conjugates analysis, HIV-infected donor T-cells were mixed with target cells (pre-labelled with CellTrace Far Red DDAO-SE,) at a 1/1 ratio and loaded on polylysine-coated coverslips (0.6×10^6^cells in 400 µl). After 1 h at 37°C, cells were fixed. For the analysis of target cells, HIV-infected donor HeLa cells were mixed with 0.5×10^6^cells target cells (pre-labelled with CellTrace Far Red DDAO-SE). After 2 hours of coculture, target cells were harvested, washed, and incubated at 37°C. At the indicated time points, target cells were loaded on polylysine-coated coverslips (0.5×10^6^cells in 400 µl), and fixed. Cells were stained with the following antibodies: rabbit anti-Gagp24 (NIH AIDS reagents program- #384), rabbit anti-Gagp24 (anti-MA, a kind gift of Pierre Boulanger), anti-tetherin (BST-2 purified MaxPab mouse polyclonal antibody B02P, Abnova or, a mouse anti-human BST2 monoclonal antibody (HM 1.24 - kindly provided by Chugai Pharmaceutical, Co.), anti-Gp120 mAb (110.H, Hybridolabs, Pasteur), FITC-conjugated cholera toxin (ChTx) subunit B (5 µg/ml, Sigma on ice). Confocal microscopy analysis was carried out with a Zeiss LSM510 microscope as described [49]. Z series of optical sections were performed at 0.2 to 0.5 µm intervals. Levels of tetherin at the cell surface were determined by flow cytometry with the HM1.24 mAb.

### Virus-target cells fusion assay

The cell-to cell viral fusion assay was adapted from a cell-free virion fusion assay [40]. HeLa donor cells were cotransfected with HIV proviruses and a plasmid carrying the Vpr gene fused with beta-lactamase (Vpr-BlaM)(a kind gift from Warner Greene) [40]. For each plasmid 1 µg was used to transfect 106 cells. After 2 h of coculture with HeLa donor cells (when stated, cocultures where gently shaken to inhibit intercellular contacts), Jurkat targets were harvested, washed and loaded with the CCF2-AM loading kit (Invitrogen) in the presence of 1.8 mM Probenecid (Sigma). Cells were incubated 2 h at room temperature then washed and fixed. The cleaved CCF2-AM fluorescence (excitation at 405 nm, emission at 450 nm) was measured by flow cytometry on a FacsCanto II (BD).

### Quantification of cell conjugates

Quantification was performed by visual observation of multiple low-power fields, directly at the microscope or after image acquisition. The total number of infected cells was counted. Cell conjugates were defined as pairs or groups of cells closely apposed among which at least one donor was Gag+. The % of infected cells with a capping of Gag or THN at the junction sites with targets was scored by visual examination.

### Quantification of Gag+ target cells

Quantification of target cells displaying large Gag+ aggregates was performed by visual observation of multiple low-power fields, directly at the microscope or after image acquisition. The total number of cells with Gag staining, and the % of cells displaying large aggregates of Gag at the cell surface were scored by visual examination. Alternatively, 3D images of the target cells were summed in the Volocity software (Perkin Elmer). Individual GagGFP+ cells were outlined manually with the ImageJ software. Viral aggregates fluorescence for each cell was measured above a 6.1% threshold and substracted of GagGFP-negative cell associated background.

### Live imaging

For live visualization of HIV movements in conjugates of infected and target cells, Jurkat cells were electroporated with pNL4-3 and pNLC4-3^EGFP^ proviral vectors or their Vpu deleted counterparts, 18–24 hours prior imaging. Cells were also cotransfected with a centrin-RFP expressing vector, to visualize the centrosome of donor cells (a kind gift of David Vaux). Electroporation of 20×10^6^ cells with 10 µg of proviral vectors, and 5 µg of centrin-RFP were performed with Gene Pulser Xcell (Bio-Rad). Jurkat cells, stably transduced with actin-RFP (actin-RFP Jurkat) were used as targets. Donors and targets were mixed in Hepes RPMI 10% FCS medium. Image acquisition began immediately after mixing cells, using a confocal spinning disk microscope in sealable microdishes (IBIDI, Germany) at 37°C. A complete 3D image was acquired every 20 seconds for 2 hours.

For the observation of viruses after transfer to targets, Jurkat-actinRFP were incubated for 4 hours at 37°C with HeLa donor cells co-transfected with pNL4-3 and pNLC4-3^EGFP^ proviral vectors or ΔVpu counterparts. Target cells were then harvested and plated on fibronectin coated sealable micro-dishes for imaging. Imaging was done at 37°C, 5% CO_2_ with a confocal spinning disk microscope (ultraView VoX, Perkin-Elmer). A complete 3D image was acquired every 5 min for 10 hours.

## Supporting Information

Figure S1 (**a**) Tetherin surface levels in Hela cells stably expressing a control shRNA (continuous line) or an shRNA targeting tetherin (Hela-THN- cells)(dotted line). (**b**) Dose response analysis of the effect of tetherin. 293T cells donor cells were cotransfected with WT (left panel) or ΔVpu (right panel) HIV proviruses (1 µg), along with the indicated doses of control (white squares) or a tetherin expression plasmid (black squares). Cells were then cocultivated with target Jurkat cells for 2 h. The percentage of Gag+ cells in targets, at different time points after harvesting the targets, is shown. Each panel is representative of 2 (20 ng and 200 ng) or 6 (100 ng) transfection experiments. The effect of tetherin on viral release was assessed by measuring the levels of Gagp24 in the supernatants of transfected cells (right panels). Results are presented as the ratio of Gag p24 in supernatants, over total levels of Gag (supernatants + cell associated p24).
(0.58 MB TIF)

Figure S2 Tetherin reduces HIV cell-to-cell transmission from CEM lymphoid cells. (**a**) Tetherin surface levels in CEM cells stably expressing a control shRNA (continuous line) or an shRNA targeting tetherin (CEM-THN- cells)(dotted line) (**b**) HIV cell-to-cell transfer. Donor CEM cells expressing (black squares) or not (white squares) tetherin (THN) were infected with HIV-ΔVpu and cocultivated with Jurkat target T cells. The appearance of Gag+ cells in targets was measured by flow-cytometry at the indicated time points (in hours). A representative experiment is shown on the left. The mean ± sd of 3 independent experiments is shown on the right (20 h time point).
(0.27 MB TIF)

Figure S3 Tetherin accumulates with Gag at the virological synapse. (**a**) Distribution of Gag and tetherin (THN) in non-infected (NI), WT or ΔVpu HIV-infected MT4C5 cells. MT4C5 cells were stained for HIV-1 Gag (green) and tetherin (red). Representative images from 6 independent experiments are shown. (**b**) Localization of Gag (green) and tetherin (red) at the virological synapse between WT or ΔVpu HIV-infected MT4C5 cells, and far-red-dye labelled Jurkat targets (blue). The Jurkat cells used in this experiment are tetherin-negative, to visualize tetherin originating from donor cells. Representative images from 5 independent experiments are shown.
(2.66 MB TIF)

Figure S4 Aspect of viral patches transferred to Jurkat cells analyzed by SEM. Correlative electron microscopy analysis of Jurkat target cells after coculture with HIV-GagGFP ΔVpu-transfected Hela donor cells. Cells are stained with anti-Env MAb coupled to 20 nm-gold particles (appearing as white dots).
(1.17 MB TIF)

Figure S5 Characteristics of viral patches transferred to Jurkat cells. WT or ΔVpu HIV-infected HeLa were cocultivated with far-red dye-labelled Jurkat cells for 2 h. Targets were then harvested and analyzed (**a**) Distribution of Gag (green) and Env (red) (**b**) Distribution of Gag (green) and cholera toxin (ChTx) (red) (**c**) Distribution of Gag (green) and tetherin (THN) (red). Representative images from at least 3 independent experiments are shown. In panel c, tetherin-negative Jurkat cells were used as targets, to visualize tetherin originating from donor HeLa cells. (ChTx-FITC was unusually pseudo-colored in red and Gagp24-Cy3 in green for the sake of clarity).
(2.49 MB TIF)

Figure S6 Tetherin reduces R5 HIV cell-to-cell transmission. (**a**) Hela donor cells expressing (black circles) or not expressing (white circles) tetherin (THN) were infected with WT (upper panel) or ΔVpu (lower panel) AD8, a R5-tropic HIV. Cells were then cocultivated with target MT4C5 cells. The percentage of Gag+ cells in targets, at different time points is shown in this experiment, representative of 2 independent ones. (**b**) Distribution of transferred WT or ΔVpu AD8 viruses on target Jurkat cells. Jurkat (which lack CCR5) cells (left panels), or primary T cells (which are CCR5+) (right panels), labelled with far-red dye (blue) were harvested after 2 h of contact with WT or ΔVpu AD8 transfected HeLa. Representative images of Gag signal on target cells are shown.
(1.21 MB TIF)

Figure S7 Tetherin reduces fusion after viral transfer to target cells. HIV fusion analyzed by cytometry, as detailed in Figure 6. Jurkat T cells were cocultivated with donor Hela cells (which are tetherin +) for 2 h, harvested and incubated at room temperature for 2 h with CCF2-AM. Viral fusion was evaluated by measuring the percentage of cells positive for cleaved CCF2-AM. When stated, cocultures were gently shaken to inhibit intercellular contacts A representative experiment (from 7 without shaking and 2 with shaking) is shown.
(0.45 MB TIF)

Video S1 Live video-microscopic imaging of cell-to-cell transfer. Jurkat cells transfected with HIV-GagGFP WT were mixed with actin-RFP expressing jurkat targets and imaged immediately. Elapsed time after mixing is indicated. A three-dimensional image was acquired every 20 seconds for 2 hours.
(4.37 MB MOV)

Video S2 Live video-microscopic imaging of cell-to-cell transfer. Jurkat cells transfected with ΔVpu were mixed with actin-RFP expressing jurkat targets and imaged immediately. Elapsed time after mixing is indicated. A three-dimensional image was acquired every 20 seconds for 2 hours.
(1.82 MB MOV)

Video S3 Live video-microscopic imaging of HIV WT on Jurkat target cells after 4 hours incubation with transfected Hela donor cells. A complete three-dimensional image was acquired every 5 minutes. Elapsed time after the beginning of acquisition is indicated.
(2.13 MB MOV)

Video S4 Live video-microscopic imaging of ΔVpu on Jurkat target cells after 4 hours incubation with transfected Hela donor cells. A complete three-dimensional image was acquired every 5 minutes. Elapsed time after the beginning of acquisition is indicated.
(1.13 MB MOV)
