# Supplementary figures and images for: Tetherin Restricts Productive HIV-1 Cell-to-Cell Transmission
Source: PLoS Pathog. 2010 Jun 17;6(6):e1000955. doi: 10.1371/journal.ppat.1000955 (PMC2887479; doi:10.1371/journal.ppat.1000955)

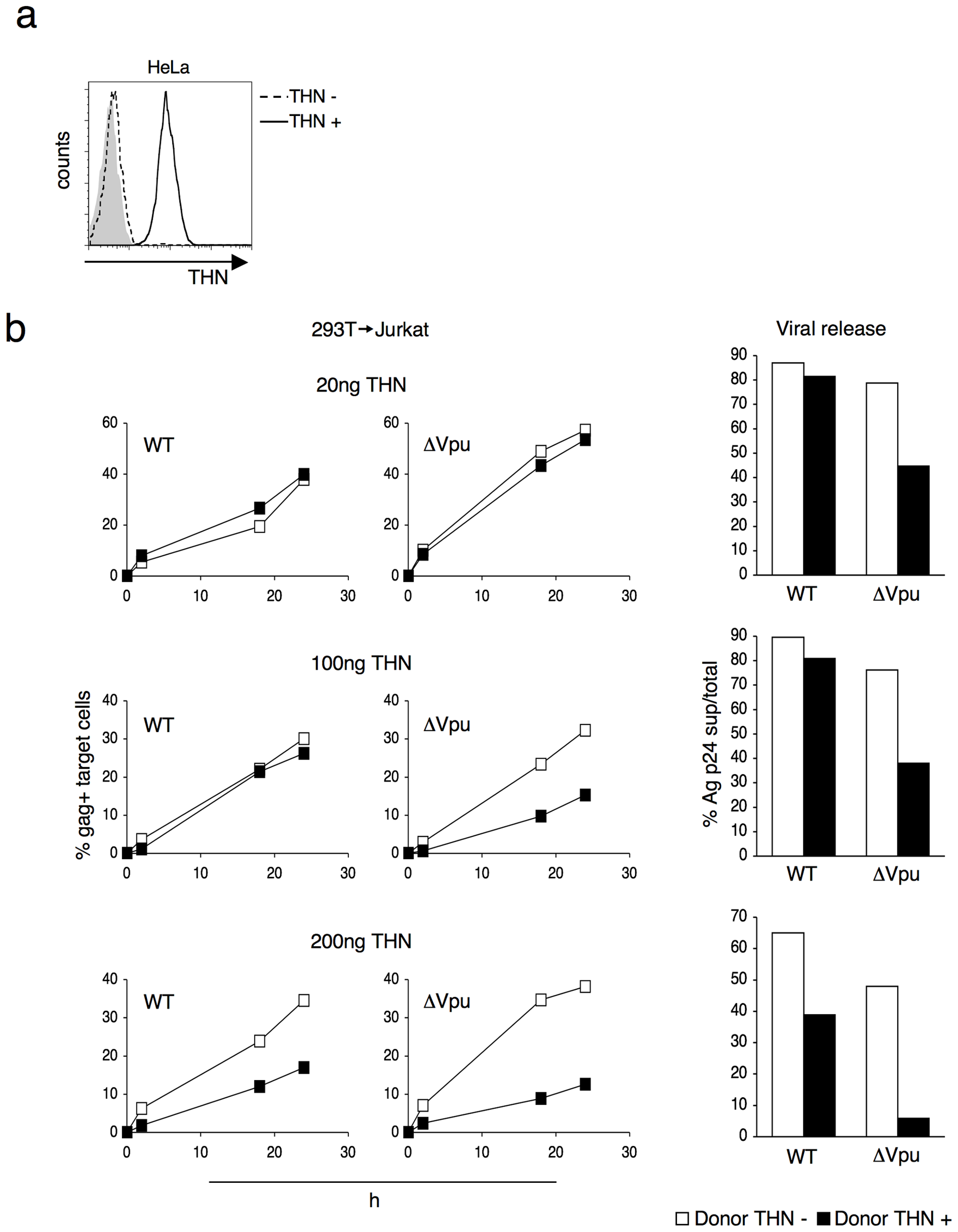

Supplement: Figure S1 — (a) Tetherin surface levels in Hela cells stably expressing a control shRNA (continuous line) or an shRNA targeting tetherin (Hela-THN- cells)(dotted line). (b) Dose response analysis of the effect of tetherin. 293T cells donor cells were cotransfected with WT (left panel) or ΔVpu (right panel) HIV proviruses (1 µg), along with the indicated doses of control (white squares) or a tetherin expression plasmid (black squares). Cells were then cocultivated with target Jurkat cells for 2 h. The percentage of Gag+ cells in targets, at different time points after harvesting the targets, is shown. Each panel is representative of 2 (20 ng and 200 ng) or 6 (100 ng) transfection experiments. The effect of tetherin on viral release was assessed by measuring the levels of Gagp24 in the supernatants of transfected cells (right panels). Results are presented as the ratio of Gag p24 in supernatants, over total levels of Gag (supernatants + cell associated p24). (0.58 MB TIF) [file ppat.1000955.s001.tif]

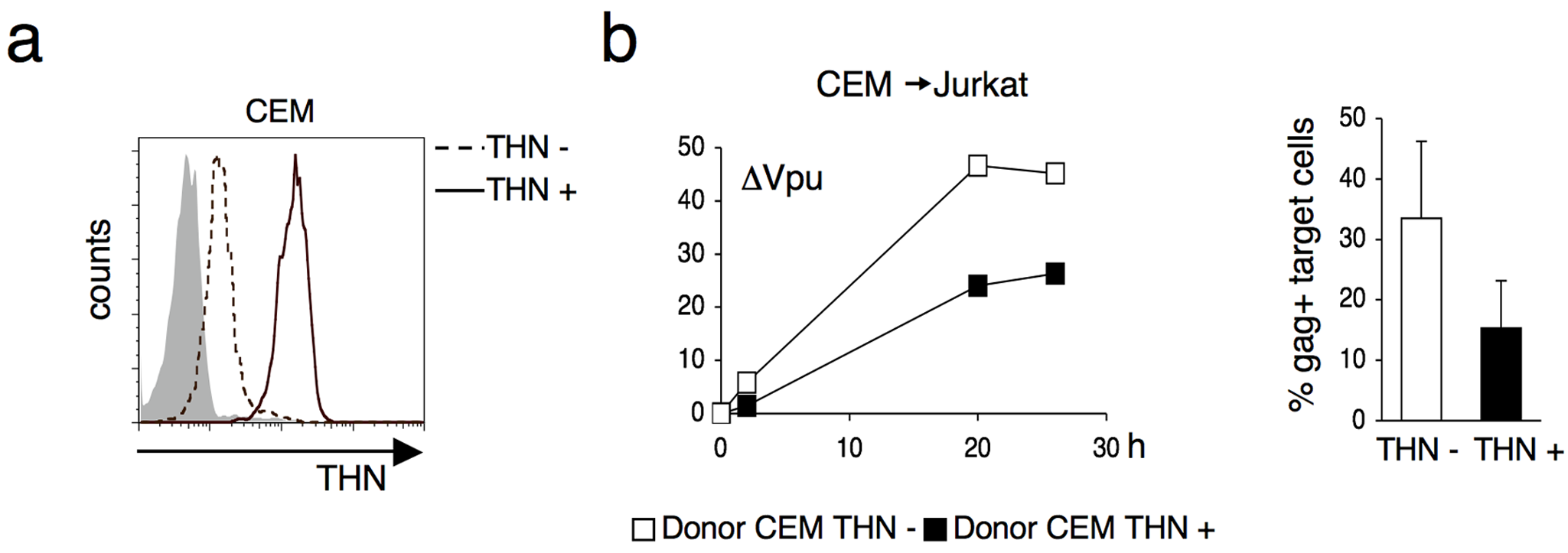

Supplement: Figure S2 — Tetherin reduces HIV cell-to-cell transmission from CEM lymphoid cells. (a) Tetherin surface levels in CEM cells stably expressing a control shRNA (continuous line) or an shRNA targeting tetherin (CEM-THN- cells)(dotted line) (b) HIV cell-to-cell transfer. Donor CEM cells expressing (black squares) or not (white squares) tetherin (THN) were infected with HIV-ΔVpu and cocultivated with Jurkat target T cells. The appearance of Gag+ cells in targets was measured by flow-cytometry at the indicated time points (in hours). A representative experiment is shown on the left. The mean ± sd of 3 independent experiments is shown on the right (20 h time point). (0.27 MB TIF) [file ppat.1000955.s002.tif]

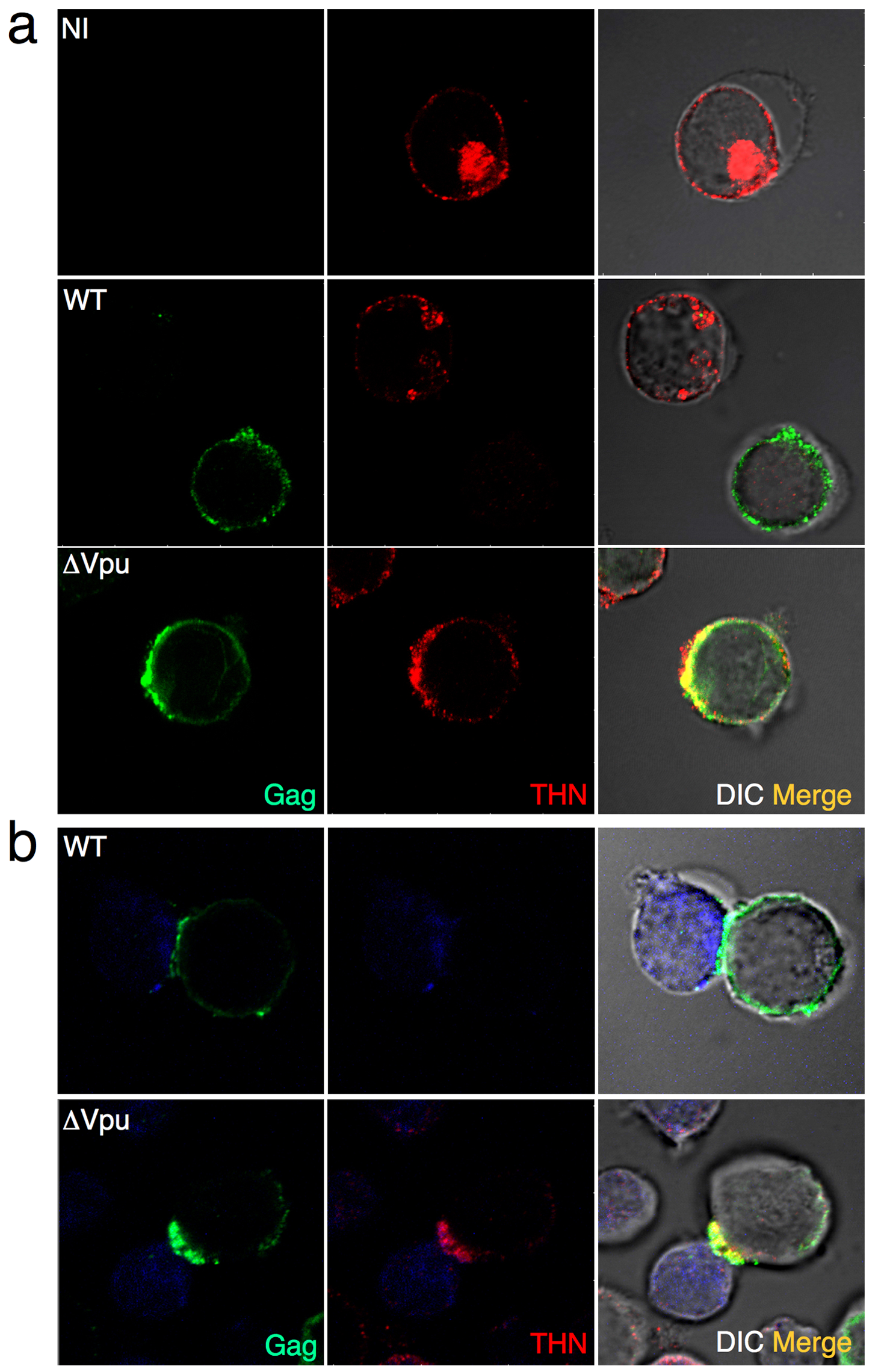

Supplement: Figure S3 — Tetherin accumulates with Gag at the virological synapse. (a) Distribution of Gag and tetherin (THN) in non-infected (NI), WT or ΔVpu HIV-infected MT4C5 cells. MT4C5 cells were stained for HIV-1 Gag (green) and tetherin (red). Representative images from 6 independent experiments are shown. (b) Localization of Gag (green) and tetherin (red) at the virological synapse between WT or ΔVpu HIV-infected MT4C5 cells, and far-red-dye labelled Jurkat targets (blue). The Jurkat cells used in this experiment are tetherin-negative, to visualize tetherin originating from donor cells. Representative images from 5 independent experiments are shown. (2.66 MB TIF) [file ppat.1000955.s003.tif]

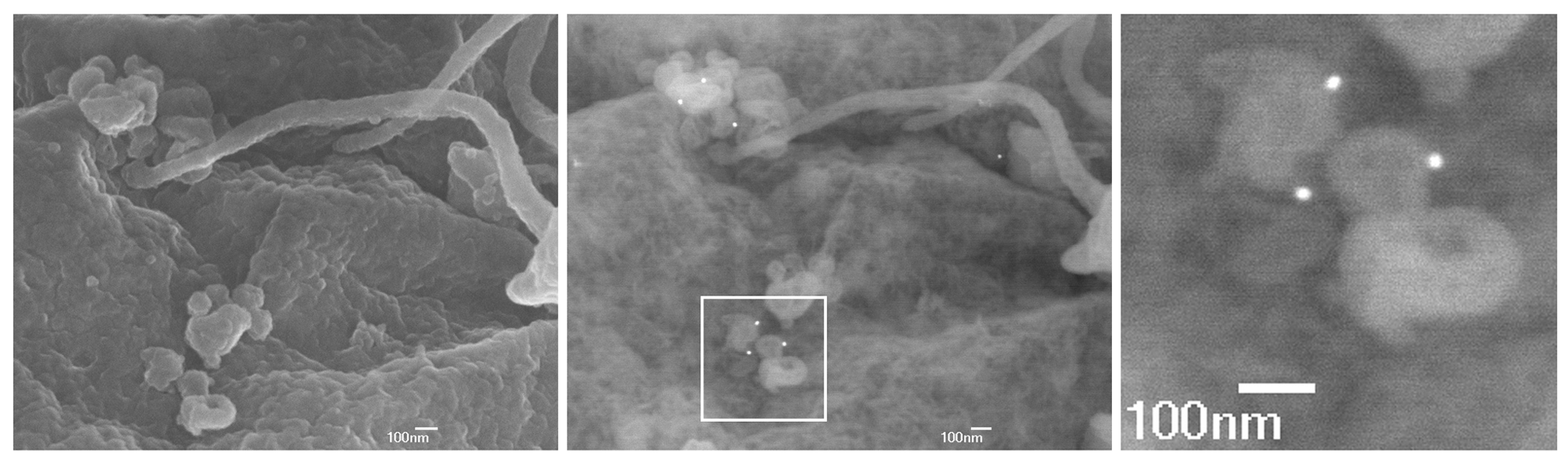

Supplement: Figure S4 — Aspect of viral patches transferred to Jurkat cells analyzed by SEM. Correlative electron microscopy analysis of Jurkat target cells after coculture with HIV-GagGFP ΔVpu-transfected Hela donor cells. Cells are stained with anti-Env MAb coupled to 20 nm-gold particles (appearing as white dots). (1.17 MB TIF) [file ppat.1000955.s004.tif]

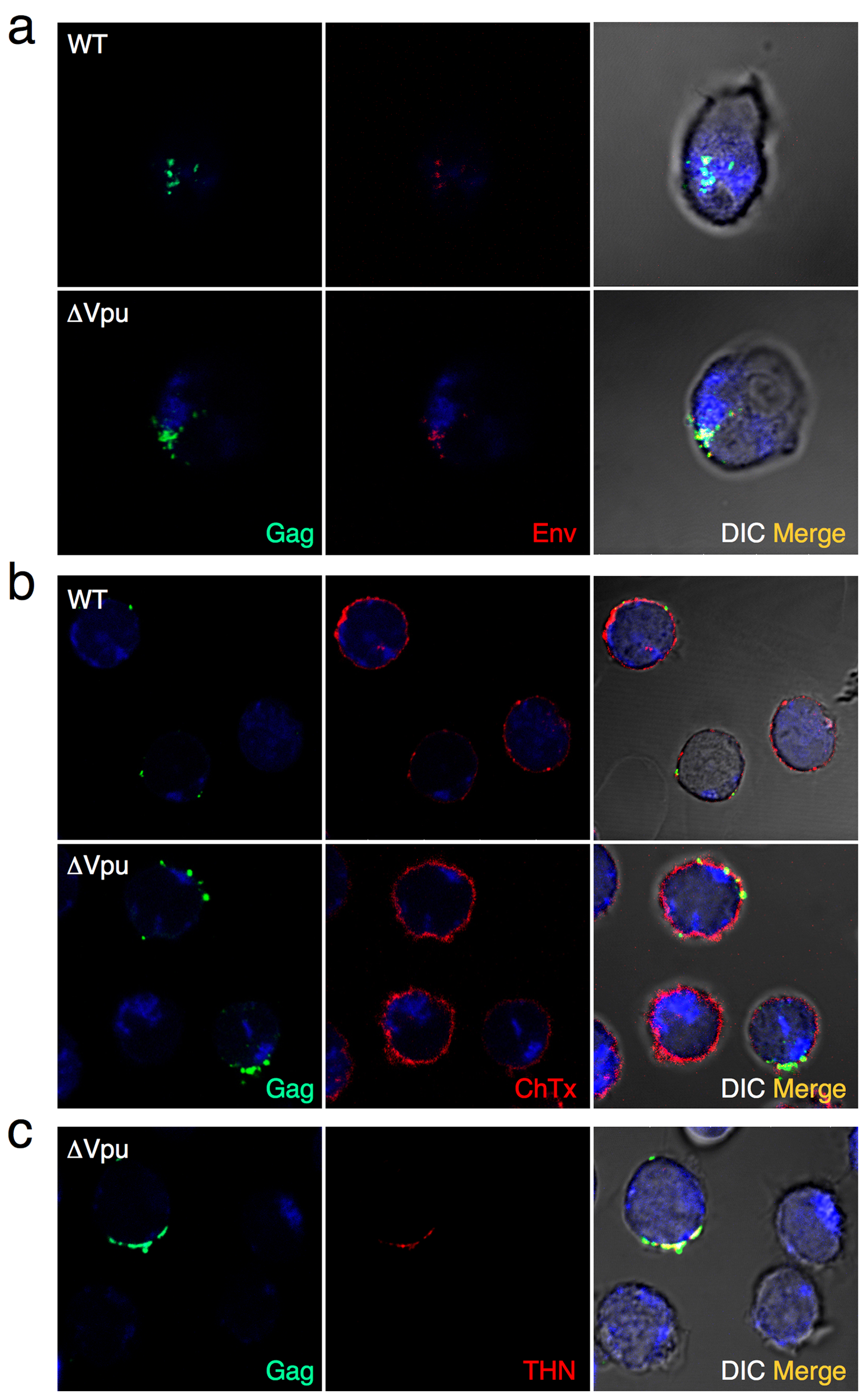

Supplement: Figure S5 — Characteristics of viral patches transferred to Jurkat cells. WT or ΔVpu HIV-infected HeLa were cocultivated with far-red dye-labelled Jurkat cells for 2 h. Targets were then harvested and analyzed (a) Distribution of Gag (green) and Env (red) (b) Distribution of Gag (green) and cholera toxin (ChTx) (red) (c) Distribution of Gag (green) and tetherin (THN) (red). Representative images from at least 3 independent experiments are shown. In panel c, tetherin-negative Jurkat cells were used as targets, to visualize tetherin originating from donor HeLa cells. (ChTx-FITC was unusually pseudo-colored in red and Gagp24-Cy3 in green for the sake of clarity). (2.49 MB TIF) [file ppat.1000955.s005.tif]

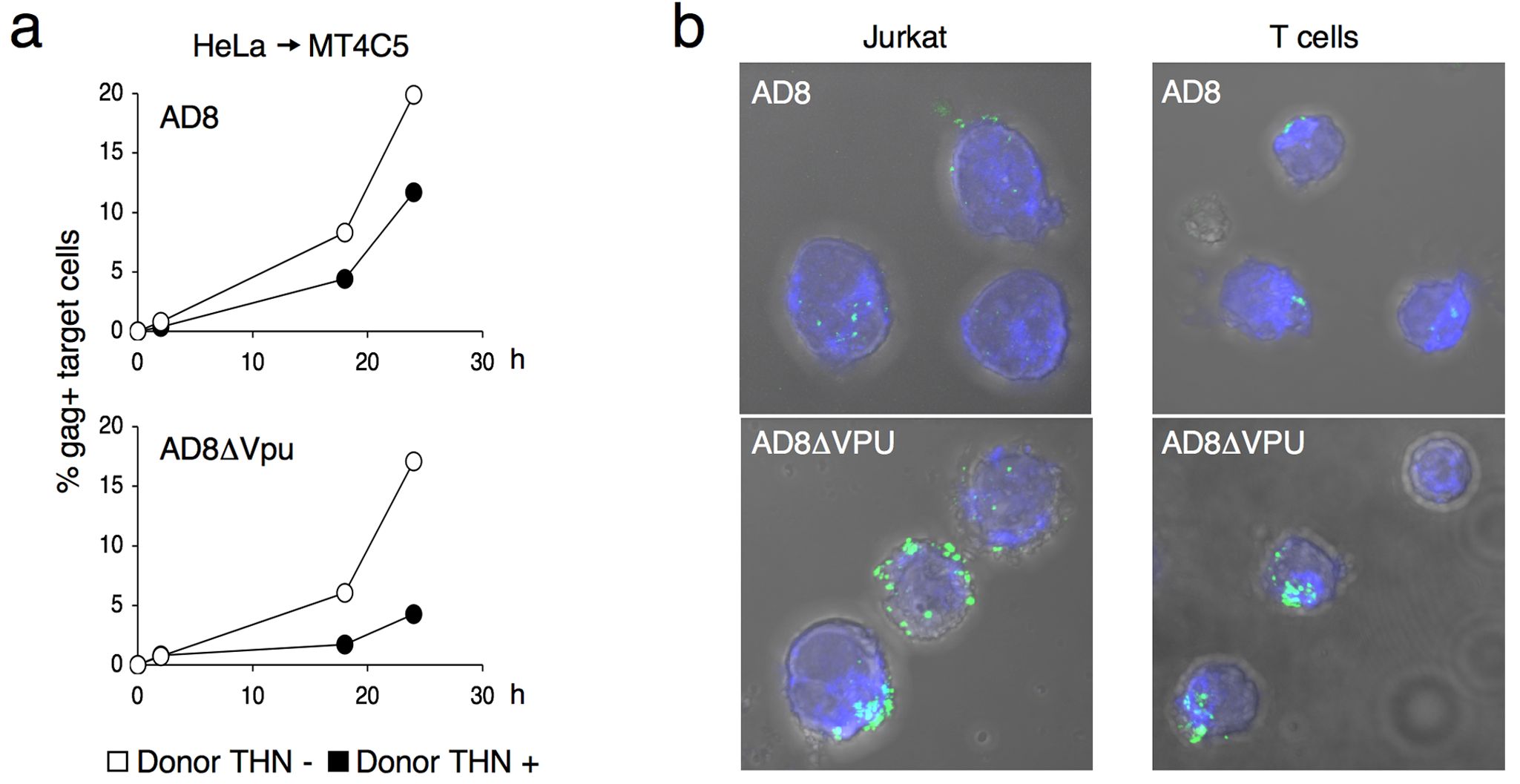

Supplement: Figure S6 — Tetherin reduces R5 HIV cell-to-cell transmission. (a) Hela donor cells expressing (black circles) or not expressing (white circles) tetherin (THN) were infected with WT (upper panel) or ΔVpu (lower panel) AD8, a R5-tropic HIV. Cells were then cocultivated with target MT4C5 cells. The percentage of Gag+ cells in targets, at different time points is shown in this experiment, representative of 2 independent ones. (b) Distribution of transferred WT or ΔVpu AD8 viruses on target Jurkat cells. Jurkat (which lack CCR5) cells (left panels), or primary T cells (which are CCR5+) (right panels), labelled with far-red dye (blue) were harvested after 2 h of contact with WT or ΔVpu AD8 transfected HeLa. Representative images of Gag signal on target cells are shown. (1.21 MB TIF) [file ppat.1000955.s006.tif]

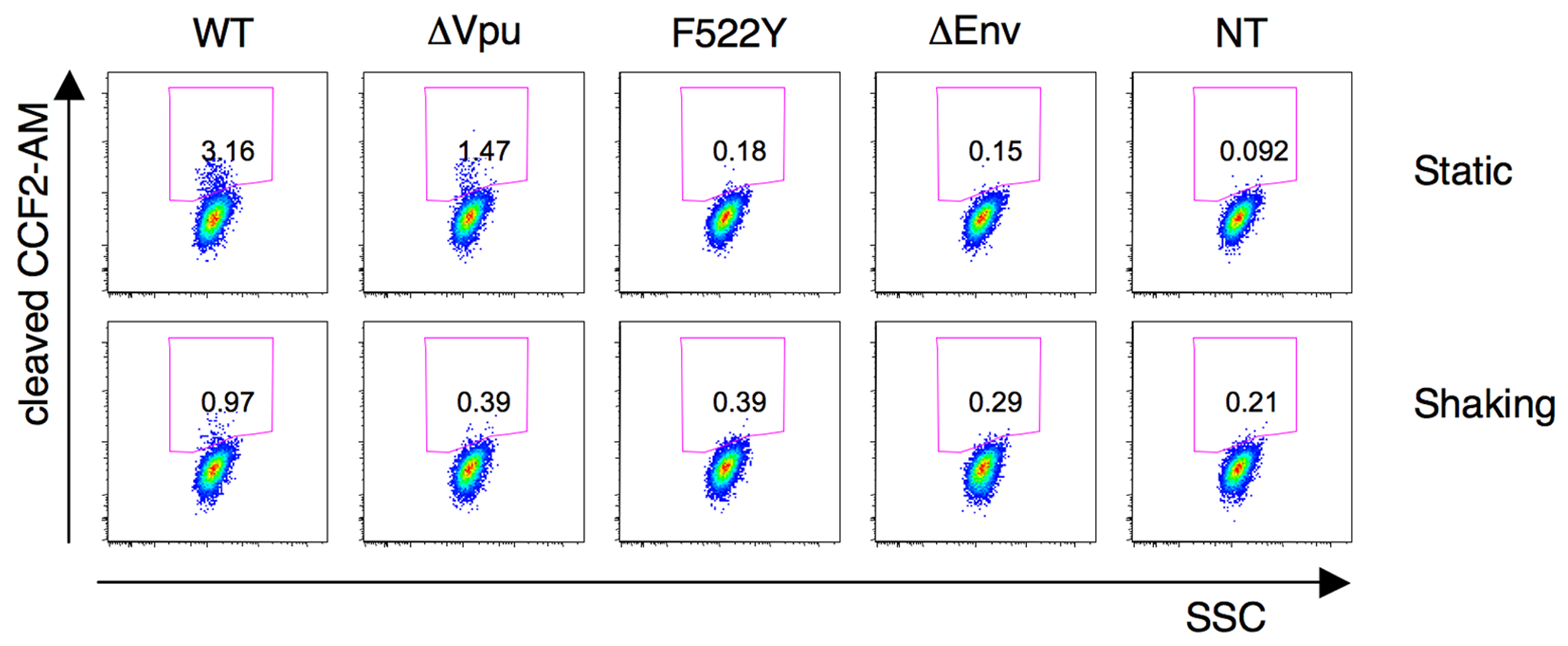

Supplement: Figure S7 — Tetherin reduces fusion after viral transfer to target cells. HIV fusion analyzed by cytometry, as detailed in Figure 6. Jurkat T cells were cocultivated with donor Hela cells (which are tetherin +) for 2 h, harvested and incubated at room temperature for 2 h with CCF2-AM. Viral fusion was evaluated by measuring the percentage of cells positive for cleaved CCF2-AM. When stated, cocultures were gently shaken to inhibit intercellular contacts A representative experiment (from 7 without shaking and 2 with shaking) is shown. (0.45 MB TIF) [file ppat.1000955.s007.tif]
